# Impact of Super-High Density Olive Orchard Management System on Soil Free-Living and Plant-Parasitic Nematodes in Central and South Italy

**DOI:** 10.3390/ani12121551

**Published:** 2022-06-15

**Authors:** Silvia Landi, Giada d’Errico, Rossella Papini, Ilaria Cutino, Stefania Simoncini, Andrea Rocchini, Giorgio Brandi, Roberto Rizzo, Giovanni Gugliuzza, Giacinto Salvatore Germinara, Salvatore Nucifora, Gaetana Mazzeo, Pio Federico Roversi

**Affiliations:** 1 Consiglio per la ricerca in agricoltura e l’analisi dell’economia agraria CREA DC–Centro di Ricerca Difesa e Certificazione, Via Lanciola 12/A, Cascine del Riccio, 50125 Florence, Italy; silvia.landi@crea.gov.it (S.L.); ilaria.cutino@crea.gov.it (I.C.); stefania.simoncini@creagov.onmicrosoft.com (S.S.); andrea.rocchini@crea.gov.it (A.R.); piofederico.roversi@crea.gov.it (P.F.R.); 2 Dipartimento Scienze Agrarie, Università di Napoli Federico II, Via Università 100, Portici, 80055 Napoli, Italy; 3 Consiglio per la ricerca in Agricoltura e l’analisi dell’economia agraria CREA AA–Centro di Ricerca Agricoltura e Ambiente, Via Lanciola 12/A, Cascine del Riccio, 50125 Firenze, Italy; papinirossella30@gmail.com (R.P.); giorgio.53@hotmail.it (G.B.); 4 Consiglio per la ricerca in Agricoltura e l’analisi dell’economia agraria CREA DC–Centro di Ricerca Difesa e Certificazione, SS113 KM 245,500, Bagheria, 90011 Palermo, Italy; roberto.rizzo@crea.gov.it (R.R.); giovanni.gugliuzza@crea.gov.it (G.G.); 5 Dipartimento di Scienze Agrarie, Alimenti, Risorse Naturali e Ingegneria, Università di Foggia, Via Antonio Gramsci 89, 71122 Foggia, Italy; giacinto.germinara@unifg.it; 6 Dipartimento di Agricoltura, Alimentazione e Ambiente (Di3A), Università di Catania, Via Santa Sofia, 100, 95123 Catania, Italy; nucifora@unict.it (S.N.); gamazzeo@unict.it (G.M.)

**Keywords:** nematode indicators, *Olea europaea*, soil management, soil physico-chemical properties

## Abstract

**Simple Summary:**

Super-high density olive orchards are spreading in Italy to reduce production costs and increase yields per hectare. The objective of this study was to assess the orchards’ effect on the soil nematode community in five sites located in the main Italian olive cultivation areas compared to the adjacent traditional olive orchard system. Super-high density olive orchard management combined with conventional tillage and mineral fertilization decreased total organic carbon compared to traditional management. The soil nematode community was affected by the depletion of organic matter, especially for plant-parasitic nematodes, which increased. Moreover, this investigation evidenced that the Super-high density olive orchard management system could change the soil plant-parasitic nematode composition of olive orchards. In fact, the families Telotylenchidae, Paratylenchidae, Meloidogynidae, and Criconematidae were favored in the Super-high density olive orchard system, while Longidoridae, Heteroderidae, and Pratylenchidae were disadvantaged. However, conservative and sustainable soil management might maintain or improve the soil nematode community functionality and reduce plant-parasitic nematodes.

**Abstract:**

The soil nematode community plays an important role in ecosystem services. The objective of this study was to assess the effect of Super-high density (SHD) olive orchards on the nematode community in five sites with different soils, climates, and cultivars. At each site, the SHD management system was compared to the adjacent olive orchard traditional (TRAD) system, in which the same soil management and phytosanitary measures were applied. Soil management was assessed by total organic carbon content (TOC), while the soil nematode community was evaluated using the nematode taxa abundances and soil nematode indicators. TOC was significantly decreased in the SHD olive orchard system compared to TRAD in the sites characterized by conventional tillage and mineral fertilization. The two-way ANOSIM analysis on nematode abundance showed no difference between the two olive management methods, instead showing only a significant difference per site mainly due to variabilities in plant-parasitic nematode assemblage. However, a negative impact of SHD management was evident in environments stressed by summer droughts and conventional tillage: the ratio of obligate plant-parasites to bacterivores and fungivores (Pp/(B+F)) was significantly higher in SHD than in the TRAD olive orchard system, and the prey-to-predator θ mass ratio showed the lowest values in the sites under organic fertilization or green manure. The canonical correspondence analysis showed that the free-living nematodes were only slightly affected by SHD olive orchards; instead, the presence of plant-parasitic nematodes families such as Telotylenchidae, Paratylenchidae, Meloidogynidae, and Criconematidae was favored, in comparison to Longidoridae, Heteroderidae, and Pratylenchidae.

## 1. Introduction

*Olea europaea* L. subsp. *europaea* is one of the oldest cultivated olive trees across the Mediterranean basin. Italy represents one of the main olive oil producers in the world, with a cultivated area of about 1.1 million hectares distributed in the Central and South of the country and with a turnover of 3% of the entire agri-food sector [1]. The production of olive oil is strongly influenced by meteorological events or pests and pathogen attacks, as well as by obsolete management and expensive production practices. To reduce production costs and at the same time increase yields per hectare, super-high density (SHD) olive orchards are gaining popularity in Italy. In SHD plantings, the concept of a single tree is replaced by a productive wall, in which integral mechanization, including pruning and harvesting, is applied. In the last years, a surface corresponding to less than 5% of the total olive orchard area was converted in the SHD olive orchard system, and the largest areas are in Apulia, Tuscany, and Sicily regions [2].

The potential impact of this change in the olive cropping system on soil biological communities, especially soilborne pathogens and pests, has been poorly investigated. Generally, in traditional olive cropping systems, the plant-parasitic nematode population levels are low in the rhizosphere [3], although *Meloidogyne* spp. may be responsible for 5–10% of crop losses [4]. Although plant-parasitic nematodes belonging to the *Meloidogyne* genus are present in Italy, studies that estimate the economic damages caused by them are missing. Besides *Meloidogyne* spp., the dominant nematode genera found in association with Italian olive orchards are *Pratylenchus*, *Helicotylenchus*, *Rotylenchus*, *Tylenchorhynchus*, *Heterodera*, and *Tylenchulus* [5,6,7,8,9]. In addition, the damages due to plant-parasitic nematodes may be synergistic when combined with other pathogens such as fungi or viruses [10,11]. In this perspective, the virus-vectors *Xiphinema* spp. and *Longidorus* spp., frequently found in the olive rhizosphere, may become a serious threat [12].

From a large-scale investigation conducted in the whole district of Andalusia, plant-parasitic nematodes seem to be slightly influenced by agronomic practices, including different densities of plant/hectare [13]. However, specific studies on the impact of SHD olive orchards on plant-parasitic nematodes and/or on the whole soil nematode community are missing. Nematodes play a key role in the soil ecosystem because they occupy all consumer trophic levels of the soil food web [14]. In the framework of ecosystem services, free-living (bacterial and fungal feeders and their predators) and plant-parasitic nematodes are directly involved in the nutrient recycling of carbon, nitrogen, and phosphorus as supporting services [15,16] and in pest regulation services, respectively [17]. An efficient regulation of these services operated by predators, including the nematodes themselves, may be useful in reducing the input of synthetic pesticides which negatively affect soil fauna and, more generally, soil health [18].

The management of SHD olive orchards includes several agronomical practices that could affect the soil nematode community, among them the selection of new cultivars characterized by small size, specific canopy management, a high irrigation requirement due to an irregular distribution of the solar radiation incidence into the canopy [19], and a high level of mechanization with heavy machines. In this regard, several authors report that the olive plant genotype may influence the soil nematode community population [20]. Moreover, other authors state that the plant-parasitic nematodes are susceptible to agronomic practices such as green cover and irrigation [21,22,23]. Finally, soil compaction caused by tillage and harvesting machines could affect the soil nematode community [17,24].

The present study aimed to assess the impact of SHD olive orchards on the soil nematode community. The SHD was compared to traditional (TRAD) olive orchard systems in five sites located across the most representative olive-producing Italian regions (Apulia, Tuscany, and Sicily) characterized by different soil types, climates, and consequently, several cultivars. This study supports the hypothesis that the investigated cultivars, the water management, and the heavy mechanization could affect the soil nematode structure. In detail, the effect of the SHD management system was assessed on: (i) change in nematode taxa abundance for free-living and plant-parasitic nematodes, (ii) efficiency of food web both for nutrient mineralization and plant-parasitic nematodes’ regulation by soil nematode indicators, and (iii) the relationship between the main soil properties and the agronomic management with the soil nematode community.

## 2. Materials and Methods

### 2.1. Field Sites

The selected experimental fields were located in Firenze (FIR) and Siena (SIR) (Tuscany, Central Italy), Foggia (FOG) (Apulia, South Italy), and Ragusa (RAG) and Trapani (TRP) (Sicily, South Italy) (Table 1; Figure S1 and Table S1). The field trials were carried out in 2018 and 2019 by using the same experimental design in each site. Two adjacent areas belonging to the same farm were selected to compare the soil nematode community between SHD and TRAD olive orchard systems. The total experimental area of each farm was 3.0 ha, consisting of two plots (1.5 ha per plot for each management). The density cultivation of the SHD and the TRAD was 1500 and 400 plants/hectare, respectively. In all sites, TRAD orchards were used for the cultivation of olive trees for over 40 years, while the SHD for approximately 5–15 years. In each site, soil and phytosanitary practices were the same under both systems.

Complementary irrigation and mechanical harvest were applied in SHD plots, except the RAG site, where regular and complementary irrigations were applied in SHD and TRAD, respectively.

### 2.2. Soil Sampling Design

In 2018 and 2019, soil samples were collected in each study area both in spring and autumn, in three different marked rows of both orchard systems for each study area. For assessment of the nematode community, samples were taken at a 0–30 cm depth after removing surface residues. Each soil sample that was obtained mixed six cores, randomly collected. To characterize the main soil chemical properties, a further set of soil samples at the same depth was collected in proximity to the previous samplings. A total of 120 samples were collected both for chemical and biological analysis corresponding to three replicates/treatment/year/season/site. Each sample was then placed in a sterile plastic bag, labeled, and stored in a cold chamber at 4 °C until analyses.

### 2.3. Soil Chemical Analysis

The soil samples were air-dried at room temperature (~20 °C) and sieved through a 2 mm mesh for texture and soil pH, and a 0.5 mm mesh for soil total organic carbon (TOC). The texture, reported in Table 1, was determined by modified pipette methods [25]. Soil pH was measured potentiometrically in a 1:2.5 soil–water suspension. TOC was determined by hot oxidation with potassium dichromate and sulfuric acid [26].

### 2.4. Soil Nematode Community Analysis

Nematodes were extracted by the cotton-wood filter method and identified to the genus or family level as described by Landi et al. [17]. Soil nematode communities and their relationships with soil properties were investigated based on the following population parameters: (i) abundance of nematode taxa at the family level, (ii) ratio of obligate plant parasites (Pp) to bacterivores (B) and fungivores (F) (Pp/(B+F)) [27], (iii) maturity index [28] and the food web indicators (BI, basal index; EI, enrichment index; SI, structure index; CI, channel index) according to Ferris et al. [14], (iv) diversity-weighted abundance based on nematode biomass (θ) and arranging soil nematode population on a functional basis into detritivores (bacterial and fungal feeders), plant-parasitic nematodes, and predators (including omnivores) [29,30], and (v) prey-to-predator θ mass ratio to evaluate regulation function [17,30].

### 2.5. Statistical Analysis

Two-way ANOVA was performed to assess the influence of system and year on soil chemical properties and nematode indicators. When the F-test was significant at *p* < 0.05, treatment means were compared using the Student–Newman–Keuls test by the CoStat statistical software package (https://www.Cohort.com/costat.html (accessed on 20 March 2021)). In addition, nematode communities were compared using multivariate methods provided by the Past analysis package [31] (https://folk.uio.no/ohammer/past (accessed on 30 March 2021)). Nematode communities were compared using analysis of similarity (ANOSIM) and Simper analysis based on the Bray–Curtis similarity index, nearest-neighbor [32]. The nematode abundance data were transformed using the square root. Bonferroni correction *p*-value was applied. Canonical correspondence analysis (CCA) was carried out to link nematode communities (abundance of nematode taxa and indicators) and environmental variables (texture, soil pH, TOC, SHD, and TRAD orchard management systems). Only the significant environmental axes were considered and are represented by vectors.

## 3. Results

### 3.1. Soil Chemical Properties

**Figure 1:**
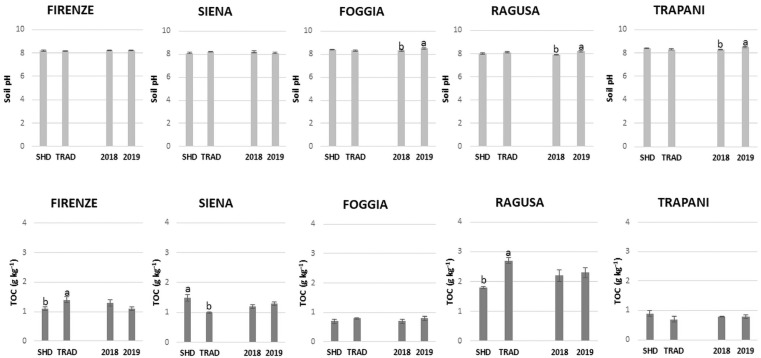
Soil chemical properties: pH and total organic carbon (TOC) of topsoil (0–30 cm) in Super-high density (SHD) and traditional (TRAD) olive orchard systems, in the five experimental sites. Different letters indicate significant differences, *p* < 0.05, Student–Newman–Keuls test.

Soil pH ranged from 8 (SHD, RAG) to 8.5 (SHD, FOG), evidencing soils with moderate alkalinity without differences between orchard systems. Although this parameter did not show relevant annual shifts, it significantly increased across the two investigated years in FOG, RAG, and TRP (Figure 1). The TOC concentration showed the lowest values in FOG and TRP sites, evidencing an environment poor in organic matter content with a range from 0.7 (SHD, FOG; TRAD, TRP) to 0.9 (SHD, TRP). Conversely, the highest values in TOC content were found in RAG (1.8–2.7 mg kg^−1^). In orchard systems, the maximum content in TOC was found in RAG and FIR sites under traditional management, and in the SIE site under SHD olive orchard systems. No significant differences were found between the two years.

### 3.2. Soil Nematode Structure

Nineteen plant-parasitic and free-living nematode families were identified in soil samples collected from the five selected sites. The two-way ANOSIM analysis on nematode abundance showed a small but significant difference per site (R = 0.28, *p* < 0.0001) and no difference per orchard system (R = 0.03, *p* < 0.16). The R values for sites FOG-FIR, FOG-SIE, FOG-RAG, and FOG-TRP pairwise comparisons were 0.29 (*p* < 0.001), 0.38 (*p* < 0.001), 0.51 (*p* < 0.001), and 0.43 (*p* < 0.001), respectively. Data showed that the FOG site was significantly different from the others. Additionally, the TRP site was significantly different from SIE and RAG sites (TRA-SIE, R = 0.41, *p* < 0.001; TRA-RAG, R = 0.33, *p* < 0.001). Small differences were found between FIR-SIE (R = 0.12, *p* < 0.03) and FIR-RAG (R = 0.16, *p* < 0.008), whereas no difference with SIE-RAG (R = 0.09, *p* < 0.13). Among these sites, the Bray–Curtis dissimilarity using SIMPER showed 46.13% of the overall dissimilarity (Table S2). Differences were mainly due to the high abundance of families Hoplolaimidae followed by Rhabditidae, Tylenchidae, and Cephalobidae. Family break-down of similarity showed that 10 families accounted for 95% of this dissimilarity. The lowest abundance and number of taxa were found in FOG and SIE sites, respectively. The trophic groups of plant-parasitic nematodes and predators showed the highest differences in taxa diversity among sites. Regarding plant-parasitic nematodes, the Hoplolaimidae family showed high abundance only in SIE and RAG sites. Moreover, the families Meloidogynidae and Criconematidae were found only in FOG and RAG sites, respectively, and the families Heteroderidae and Longidoridae were present only in the FIR site. Concerning predators, the Discolaimidae family was found only in FIR and RAG sites, while Seinuridae only in the RAG site.

Although no global difference was found between SHD and TRAD olive management systems, the site-by-site data evaluation, based on similarity analysis on taxa nematode abundance, evidenced a spatial separation between SHD and TRAD in FOG for the whole soil nematode community (R = 0.20, *p* < 0.001) and in RAG only for plant-parasitic nematodes (R = 0.11, *p* < 0.05). The SIMPER analysis confirmed 49.37% of overall average dissimilarity in the FOG site, in which 11 families accounted for 95%, and differences were mainly due to the high abundance of Rhabditidae and Cephalobidae in TRAD compared to the SHD management system (Table S3). Instead, SIMPER analysis performed on the RAG site showed 47.35% of dissimilarity, in which only three families, Hoplolaimidae, and to lesser extent Telotylenchidae and Praylenchidae, accounted for 95%. Specifically, Hoplolaimidae was more abundant in SHD, while Telotylenchiae and Pratylenchidae were prominent in TRAD olive management (Table S4).

### 3.3. Soil Nematode Indicators

The averages of MI, PPI, BI, EI, SI, CI, and Pp/(B+F) values for each management and year are reported in Table 2.

Few significant differences were found between orchard systems: the BI values in FOG were significantly higher in TRAD than in the SHD site, the CI values in FOG were higher in SHD than TRAD, and the Pp/(F+B) values in FOG and RAG sites showed significant increments in SHD olive orchards compared to TRAD. Instead, in most cases, the community indices exhibited a significant annual shift. In fact, MI and SI increased across the two monitoring years in all sites, while BI decreased. In three sites (SIE, RAG, and TRP), PPI showed a significantly higher value in 2019 than in 2018. Only the CI was never influenced by year.

**Figure 2:**
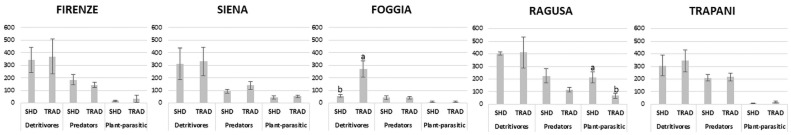
Diversity-weighted abundance (Ɵ) for functional classes of soil assemblages in five Italian sites. SHD, Super-high density olive orchards; TRAD, traditional olive orchards. Different letters indicate significant differences at *p* < 0.05. Standard errors are reported.

Average values the of diversity-weighted abundance (θ) index expressed as biomass are reported in Figure 2.

Overall, the dominant functional class in all sites and each orchard system was represented by detritivores (bacterial and fungal feeders), except for SHD in FOG, in which a significant decrease was found. Instead, plant-parasitic nematodes were generally low, except for RAG, in which the highest value was found in the SHD management. The predator channel (including omnivores) ranged from 42.3 ± 15.4 (FOG-SHD) to 215.6 ± 28.5 (TRP-TRAD), and no significant differences were evidenced between the two olive orchard systems amongst all sites. Overall, the prey-to-predator θ mass ratio was generally high, indicating an inadequate regulatory function of predators against opportunistic and plant-parasitic nematodes. The TPR site exhibited the lowest prey-to-predator θ mass ratio in SHD (1.5) and TRAD (1.7), indicating the best regulation among the selected sites. The highest differences between management methods were found in the FOG site, in which this ratio ranged from 1.5 to 6.7 in SHD and TRAD, respectively. In the other sites, the ratio of prey/predator was approximately similar and ranged from 2 in FI-SHD to 3.8 in SI-SHD.

### 3.4. Relationship among Environmental Variables and Nematode Community Structure

**Figure 3:**
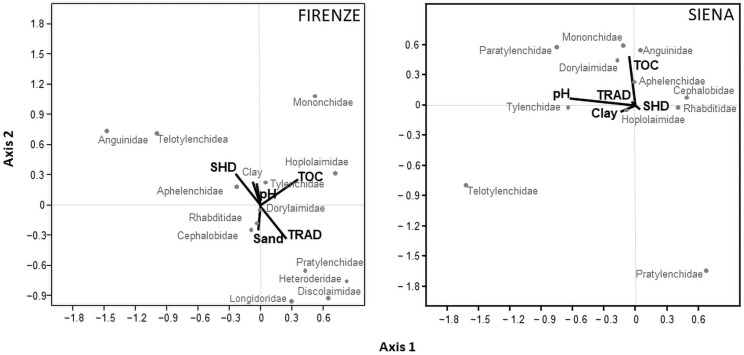
Scatter plots of CCA between soil properties and nematode taxa abundance. Firenze, percentage of variance explained was 37.50% for axis 2 (*p* < 0.04), and no significance for axis 1. Siena, percentage of variance was 79.77% for axis 1 (*p* < 0.02) and 24.05% for axis 2 (*p* < 0.03).

**Figure 4:**
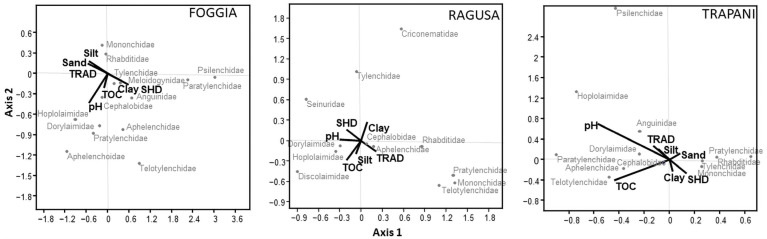
Scatter plots of CCA between soil properties and nematode taxa abundance. Foggia, percentage of variance was 43.50% for axis 1 (*p* < 0.05) and 38.67% for axis 2 (*p* < 0.001). Ragusa, percentage of variance explained was 32.61% for axis 2 (*p* < 0.05), and no significance for axis 1. Trapani, percentage of variance was 48.99% for axis 1 (*p* < 0.01) and 33.83% for axis 2 (*p* < 0.001).

The CCA, conducted between nematode taxa and environmental variables, showed that both olive orchard systems influenced the abundance of nematode taxa in four sites, especially for plant-parasitic nematodes and predators (Figure 3 and Figure 4). Instead, in the SIE site, the soil variables were dominant compared to the orchard system features in the nematode community only. Moreover, the dominant families of free-living nematodes such as Rhabditidae, Cephalobidae, and Dorylaimidae were poorly affected by these variables.

In the FIR site, axis 2 was dominated by SHD (0.31), TRAD (−0.31), and TOC (0.25). The families of Mononchidae and Discolaimidae were mainly influenced by soil properties such as TOC and sand, and to a lesser extent by SHD and TRAD management methods. Instead, the plant-parasitic nematodes were mainly influenced by management. The families of Anguinidae and Telotylenchidae were positively related to SHD, while the families of Pratylenchidae, Heteroderidae, and Longidoridae favored TRAD management (Figure 3). In the SIE site, axes 1 and 2 were dominated by soil pH (−0.64) and TOC (0.49), respectively, and the families of Pratylenchidae and Telotylenchidae were inversely related to TOC (Figure 3). In the FOG site, axis 1 was driven by clay (0.59), sand (−0.57), silt (−0.51), soil pH (0.52), SHD management (0.39), and TRAD (−0.39), while axis 2 was dominated by soil pH (−0.43). Significant community variables are reported as follows: (1) the plant-parasitic families Psilenchidae, Paratylenchidae, and Meloidogynidae were favored by the SHD system, (2) the families of Hoplolaimidae, Dorylaimidae, Pratylenchidae, and Aphelenchoidae were driven by soil pH, and (3) the families Telotylenchidae and Aphelenchidae were related to SHD management and soil pH (Figure 4). In the RAG site, axis 2 was dominated by TOC (−0.29) and clay (0.27), and Criconematidae and Tylenchidae families were favored by clay, and to a lesser extent by SHD management. Telotylenchidae and Mononchidae families were related to TOC and TRAD management (Figure 4). In the TRP site, axis 1 was dominated by soil pH (−0.57) and TOC (−0.44), and axis 2 by soil pH (0.70), TOC (−0.40), SHD management (−0.27), and TRAD management (−0.27). Finally, the families Hoplolaimidae, Psilenchidae, and Paratylenchidae were favored by soil pH, and to a lesser extent by TRAD management (Figure 4).

**Figure 5:**
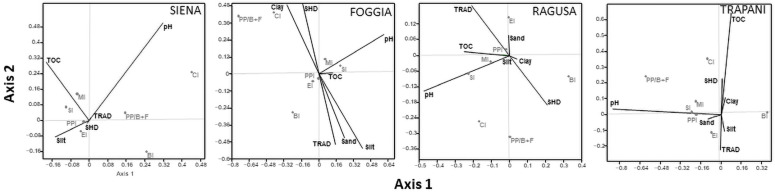
Scatter plots of CCA between soil properties and nematode indicators (MI, maturity index; PPI, plant-parasitic index; BI, basal index; CI, channel index; EI, enrichment index; SI, structure index; Pp/B+F, the ratio of obligate plant parasites to bacterivores and fungivores). Siena, percentage of variance explained was 29.35% for axis 2 (*p* < 0.05), no significance for axis 1. Foggia, percentage of variance was 57.49% for axis 1 (*p* < 0.04) and 36.75% for axis 2 (*p* < 0.004). Ragusa, percentage of variance explained was 24.38% for axis 2 (*p* < 0.05), no significance for axis 1. Trapani, percentage of variance was 80.91% for axis 1 (*p* < 0.001) and 18.00% for axis 2 (*p* < 0.007).

The biplot of CCA conducted between soil nematode indicators and soil environmental variables showed that the indices MI, PPI, EI, and SI were the least influenced by the environmental gradient established within the study plots (Figure 5). In the FIR site, no significant differences were found. In the SIE site, axis 2 was dominated by soil pH (0.50) and TOC (0.30); the CI, which was plotted furthest from the origin and therefore varied most within the environmental gradient, was positively correlated with soil pH and inversely with TOC. In the FOG site, axis 1 was dominated by soil pH (0.60), silt (−0.40), and clay (−0.29), while axis 2 by silt (−0.53), clay (0.49), sand (−0.44), SHD management (0.50), and TRAD management (−0.50). The CI and Pp/(B+F) were driven by clay and SHD management. In the RAG site, axis 2 was dominated by SHD (−0.20) and TRAD (0.20) management. The CI and Pp/(B+F) were positively related to SHD management and soil pH. In the TRP site, axis 1 was dominated by soil pH (0.85), while axis 2 by TOC (0.64) and to a lesser extent by SHD (0.23) and TRAD (−0.23) management. Furthermore, the Pp/(B+F) was driven by soil pH, CI was favored by TOC and to a lesser extent by SHD management, and finally, BI was inversely related to soil pH.

## 4. Discussion

The investigation of the most representative olive-producing Italian sites exposed to different climatic conditions and soil types allowed us to evaluate a broad variety of environments. Moreover, the selection of different experimental fields on the same farm afforded the opportunity to compare only SHD and TRAD olive orchard system features, such as different plant genotypes, smaller size, water irrigation, and the use of heavy machines for harvesting. At the same time, the soil management normally used in each farm was maintained in both areas of each site and included a broad variety of strategies, such as conventional tillage, green cover, green manure, and mineral and organic fertilization.

### 4.1. Effect of the SHD Olive Orchard Systems on Soil Fertility

In general, soil pH values were similar in each site and indicated a sub-alkaline soil environment. Despite that the soils were differently classified for texture among the sites, the clay content was high, and this created uniformity in the range of pH. Regarding TOC, these soils were poor in organic matter, especially FOG and TPR sites where organic carbon was below 1%. Only in the RAG site did the topsoil organic carbon reach the critical threshold for soil quality in temperate regions fixed at 2% [33]. However, in this site, a consistent decrease in TOC content was found in SHD olive orchards, and the same trend, although with minor intensity, was confirmed in FIR. As reported by Francaviglia et al. [34], the depletion in TOC is more marked in more intensive cropping systems such as high plant density per hectare and conventional tillage application, especially when the climatic conditions are extreme, such as dry summers without rains and high temperatures. Instead, as reported by Vignozzi et al. [35], natural grass cover and the shredding of pruning were effective in maintaining or improving TOC content in SHD olive orchard systems in the SIE site characterized by medium rainfall.

### 4.2. Effect of SHD Olive Orchard Systems on Soil Nematode Community Structure

Overall, the similarity analysis evidenced that the composition of the whole nematode community showed no relevant change in SHD compared to TRAD, while more variations were revealed for sites. However, although the sites were in two diverse climatic areas, Cfa and Csa by the Köppen Climate Classification, the differences in the soil nematode community were few, and mainly involved plant-parasitic nematodes and fewer predators. Rainfall was the climatic parameter that most affected the soil nematode community [36]. Nevertheless, as reported by other studies, climatic conditions slowly affect nematode distribution, rather than the plant species, together with soil type and soil management [17,37]. In agreement with Palomares-Rius et al. [22], soil management played a significant role to shift the structure of the nematode population in olive orchards, especially by tillage and fertilization. The site located in FOG characterized by low rainfall and with conventional soil management, such as tillage and mineral fertilization, consistently differed from other sites, showing the lowest nematode abundance and the dominance of colonizer species belonging to Rhabditidae. Instead, the SIE and RAG sites characterized by medium and low rainfall, respectively, showed no difference in the soil nematode community, probably because of the organic fertilization applied in both sites. It is well-known that organic matter is a key factor in soil biology, and several studies demonstrate that organic matter also improves the composition of nematode populations in dry soils due to its water-retention capacity [35,38,39,40].

Plant-parasitic nematode communities were peculiar to each site. Although the common genera found in the Mediterranean basin are *Criconemoides*, *Helicotylenchus*, *Longidorus*, *Meloidogyne*, *Pratylenchus, Rotylenchus*, *Tylenchorhynchus*, *Tylenchus*, and *Xiphinema* [12], their distribution and dominance were not homogeneous among the studied sites. Probably, a key role could be played by the host–parasite interaction, a factor currently scarcely investigated. Palomares-Rius et al. [20] found that nematode community populations in the rhizosphere of cultivated olive differed according to the plant genotype.

Only two sites located in south Italy showed differences between SHD and TRAD olive orchard systems in nematode taxa abundance for free-living and plant-parasitic nematodes. The soil management based on tillage might have changed the free-living and plant-parasitic nematode assemblage, respectively, in FOG and RAG sites. Conventional soil management, in soil poor in organic matter, may have caused a strong reduction of bacterial feeders belonging to Rhabditidae and Cephalobidae families in SHD compared to TRAD. Instead, in agreement with other studies, the reduction of organic matter content in soil may have increased the plant-parasitic nematode abundance in SHD, especially hoplolaimids [17,39,41].

In general, MI values indicated the presence of generalist and opportunistic species, especially where mineral fertilization together with tillage were applied. PPI showed values quite similar to those reported by Palomares-Rius et al. [20], indicating a disturbance of fields, and the highest values were found in the RAG site for both management methods due to the high abundance of hoplolaimids. The EI and SI evidenced characteristic values of perennial crops, indicating moderate disturbance, N-enrichment, and maturing food web conditions [14]. Finally, CI and EI food web indicators suggested the dominance of bacterial decomposition channels. However, soil nematode indicators also suggested that SHD may impact the soil nematode structure in the environments more stressed by climatic conditions and in which a conventional soil management system was applied. In particular, the low rainfall together with tillage application might have changed the soil nematode structure in SHD olive orchards in FOG and RAG. In the FOG site, the BI and CI showed an opposite response, in which the former decreased in SHD, while the latter increased. In fact, the detritivores channel decreased, especially for bacterial feeders belonging to the *Rhabditis* genus in SHD. Finally, the Pp/(B+F) ratio increased in SHD management in FOG and RAG sites, evidencing an increase of obligate plant-parasitic nematodes compared to detritivores, especially in the RAG site, where the plant-parasitic channel strongly increased in SHD. In contrast, more sustainable soil management such as green manure probably avoided the negative effects on the soil nematode community in the TRP site, and this site was also characterized by severe drought. Moreover, at this site, better prey/predator regulation was evident. In most cases, the community indices exhibited an annual shift, especially MI, SI, and BI. This trend was emphasized for SI in FOG and RAG due to the summer drought associated with tillage usage.

### 4.3. Soil and Management Factors Influencing Soil Nematode Structure

The soil nematode community associated with olive orchards was affected by soil physico-chemical properties in accordance with Palomares-Ruis et al. [22]. Chemical properties explored in this study (TOC and soil pH) were the factors that most influenced the soil nematode structure, more so than physical properties (texture). However, the SHD and TRAD olive orchard systems also affected nematodes, and the plant-parasitic nematode community especially. In accordance with Ali et al. [12], as expected, plant-parasitic nematodes were low among sites, and they differently responded in each site. In general, the plant parasites were mainly affected by the conditions created in the SHD management system in the sites in which conventional tillage was applied; instead, they were mainly influenced by chemical properties in the sites under green cover and green manure. Specifically, the SHD olive management system favored Telotylenchidae in FIR and FOG, Paratylenchidae and Meloidogynidae in FOG, and Criconematidae in RAG; in contrast, Longidoridae, Heteroderidae, and Pratylenchidae were not favored in FIR. These data agree with other studies, such as Ali et al. [42], who found that the intensification of agricultural practices favored the plant-parasitic nematodes, especially *Meloidogyne* spp., while the genera *Xiphinema* and *Heterodera* were prominent in wild olive trees. In terms of soil properties, the high values of soil pH also played an important role in favoring Telotylenchidae in FIR and FOG, and Hoplolaimidae and Paratylenchidae in FOG and TRP. Instead, TOC did not favor plant-parasitic nematodes, especially Telotylenchidae and Paratylenchidae, only in the SIE and RAG sites characterized by higher TOC content than other sites. Considering free-living nematodes, the most abundant families such as Rhabditidae and Cephalobidae were not influenced by the parameters explored in this study. In fact, these taxa are widely distributed across the world and well-adapted to every latitude and altitude [43]. Instead, the predators belonging to the families Mononchidae and Discolaimidae were mainly favored by high organic matter content, while Dorylaimidae (the common omnivores found in these sites) and Aphelenchoidae (fungal feeder family only found in the FOG site) were driven by soil pH [17].

Weak correlations were found between nematode community indices and environmental variables. Only CI and Pp/(B+F) were more sensitive to changes within the agronomic environments studied. According to previous studies, CI was mainly influenced by soil properties such as pH and TOC [17] in most of the sites, while Pp/(B+F) was positively related to the SHD olive orchard system in FOG and TRP sites. Moreover, the low correlations found for SI and PPI suggest that TOC content does not always improve the nematode community structure and suppresses the plant-parasitic nematodes. However, organic matter content plays a key role to regulate the ratio between obligate parasites and detritivores nematodes. In fact, the TOC below 1% found in the FOG and TRP sites favored the positive correlation between Pp/(B+F) and SHD.

## 5. Conclusions

This investigation, performed on a national scale, showed that the SHD olive orchard system may change the soil nematode community associated with olive orchards, especially concerning plant-parasitic nematodes. However, further studies are necessary to better understand the importance of the impact of management on the soil nematode community. The high plant density per hectare, the different cultivars, characterized by a smaller size than the traditional ones, and the high-water inputs seem to favor the nematodes of the families Telotylenchidae, Paratylenchidae, Meloidogynidae, and Criconematidae over Longidoridae, Heteroderidae, and Pratylenchidae. The negative effects were mainly evident in stressed environments due to the dry summers and the lowest TOC content. Nevertheless, using a conservative and sustainable soil management method may maintain or improve the soil nematode community functionality and prevent the plant-parasitic nematode increase.

## Figures and Tables

**Table 1:** Geographical position, climate parameters, soil texture, and olive orchard features of the experimental sites.

Sites	Regions	Geographical Position		Climate Parameters			Soil Texture USDA	Olive Orchard Features	
		Coordinates	Altitude a.s.l (m)	KöppenClimate Types	Mean Air Temperature (°C)	Mean Annual Precipitation (mm)		Olive Tree Cultivar	Soil Management
Firenze (FIR)	Tuscany	43.800183 N; 11.403579 E	260	Cfa	14.7	940	Clay	SHD: Leccio del Corno, Tosca, Diana TRAD: Frantoio, Leccino, Moraiolo	Conventional tillage Mineral fertilization
Siena (SIE)	Tuscany	43.275739 N; 11.603974 E	280	Csa	14.5	880	Silty Clay Loam	SHD: Frantoio, Leccino, Moraiolo, Pendolino, Leccio del Corno, Correggiolo, Maurino selezione Vittoria TRAD: Frantoio, Leccino, Moraiolo, Pendolino	Green cover Organic fertilization
Foggia (FOG)	Apulia	41.5653940 N; 15.736466 E	582	Cfa	14.5	715	Clay Loam	SHD: Arbequina TRAD: Ogliarola Garganica	Conventional tillage Mineral fertilization
Ragusa (RAG)	Sicily	37.0786790 N; 14.665837 E	380	Csa	17.0	541	Clay Loam	SHD: Tonda Iblea, Nocellara dell’Etna, Biancolilla, Moresca, Nocellara del Belice, Arbequina, Picual TRAD: Tonda Iblea	Conventional tillage Organic fertilization
Trapani(TRP)	Sicily	37.972381 N; 12.683364 E	220	Csa	17.6	680	Clay Loam	SHD: Arbequina TRAD: Biancolilla, Cerasuola, Nocellara del Belice	Green manure Organic fertilization

Cfa—Temperate, no dry season, hot summer; Csa—Temperate, dry summer, hot summer.

**Table 2:** Effect of different management methods on soil nematode indices. SHD, Super-high density olive orchards; TRAD, traditional olive orchards; MI, maturity index; PPI, plant-parasitic index; BI, basal index; EI, enrichment index; SI, structure index; CI, channel index; Pp/B+F, the ratio of obligate plant parasites to bacterivores and fungivores. Different letters indicate significant differences at *p* < 0.05. Standard errors are reported. Significant differences are highlighted in bold.

	Management		Year		Significant Effects		
	SHD	TRAD	2018	2019	M *	Y **	M + Y
**Firenze**							
MI	2.0 ± 0.1	2.1 ± 0.1	1.9 ± 0.1 b	2.3 ± 0.1 a	0.48	**0.02**	0.47
PPI	2.5 ± 0.1	2.5 ± 0.1	2.4 ± 0.1	2.5 ± 0.1	0.94	0.27	0.25
BI	37.7 ± 7.3	39.1 ± 10.6	55.2 ± 9.7 a	21.5 ± 4.6 b	0.90	**0.006**	0.35
EI	79.1 ± 2.1	77.8 ± 2.5	82.2 ± 2.2 a	74.7 ± 1.9 b	0.66	**0.02**	1.00
SI	62.9 ± 6.4	69.8 ± 3.5	59.9 ± 6.4 b	72.8 ± 2.6 a	0.34	**0.08**	0.83
CI	12.3 ± 2.5	10.4 ± 2.5	13.3 ± 3.0	9.4 ± 1.5	0.59	0.28	0.44
Pp/(B+F)	0.3 ± 0.1	0.3 ± 0.1	0.3 ± 0.1	0.3 ± 0.1	0.81	**0.02**	0.20
**Siena**							
MI	2.0 ± 0.2	2.2 ± 0.12	1.8 ± 0.1 b	2.5±0.1 a	0.33	**0.0006**	0.30
PPI	2.7 ± 0.1	2.7 ± 0.1	2.6 ± 0.1 b	2.8±0.04 a	0.99	**0.007**	0.63
BI	19.7 ± 4.7	22.4 ± 5.6	32.3 ± 5.3 a	9.8±1.4 b	0.65	**0.0008**	0.54
EI	79.9 ± 3.6	79.0 ± 4.2	82.0 ± 5.0	76.9 ± 2.0	0.88	0.38	0.76
SI	67.9 ± 5.5	72.1 ± 5.2	59.8 ± 5.6 b	80.1 ± 2.8 a	0.52	**0.005**	0.62
CI	10.4 ± 3.9	10.6 ± 4.2	15.0 ± 5.4	6.0 ± 0.8	0.97	0.13	0.81
Pp/(B+F)	0.9 ± 0.2	1.7 ± 0.5	0.9 ± 0.2	1.7 ± 0.5	0.11	0.13	**0.007**
**Foggia**							
MI	1.8 ± 0.1	1.7 ± 0.1	1.5 ± 0.06 b	2.1 ± 0.1 a	0.13	**0.00001**	**0.01**
PPI	2.0 ± 0.3	2.1 ± 0.2	2.2 ± 0.1	1.8 ± 0.3	0.79	0.3	0.30
BI	8.7 ± 1.1 b	14.5 ± 2.6 a	13.9 ± 2.6 a	9.3 ± 1.5 b	**0.01**	**0.04**	**0.0005**
EI	81.6 ± 2.4	87.8 ± 2.4	87.6 ± 2.1	81.8 ± 2.7	0.06	0.08	0.16
SI	54.4 ± 6.0	57.3 ± 6.3	40.3 ± 2.9 b	71.4 ± 4.9 a	0.62	**0.00001**	0.64
CI	12.5 ± 2.1 a	5.6 ± 2.2 b	9.6 ± 2.2	8.5 ± 2.5	**0.02**	0.71	**0.04**
Pp/(B+F)	0.2 ± 0.07 b	0.06 ± 0.03 a	0.2 ± 0.1	0.1 ± 0.04	**0.05**	0.57	0.10
**Ragusa**							
MI	2.2 ± 0.2	2.1 ± 0.1	1.9 ± 0.1 b	2.4 ± 0.1 a	0.72	**0.006**	0.60
PPI	2.9 ± 0.06	2.8 ± 0.1	2.7 ± 0.1 b	3.0 ± 0.03 a	0.50	**0.02**	0.98
BI	61.7 ± 18.5	37.7 ± 7.0	79.6 ± 15.7 a	19.9 ± 2.9 b	0.12	**0.0007**	0.12
EI	72.7 ± 4.9	78.7 ± 3.8	74.6 ± 5.3	76.9 ± 3.4	0.37	0.73	0.93
SI	60.0 ± 7.5	66.1 ± 7.0	46.8 ± 6.7 b	79.3 ± 3.8 a	0.45	**0.0006**	0.84
CI	9.4 ± 4.4	6.9 ± 1.7	10.4 ± 4.3	6.0 ± 1.7	0.59	0.36	0.18
Pp/(B+F)	1.9 ± 0.4 a	1.0 ± 0.4 b	1.0 ± 0.3 b	2.0 ± 0.4 a	**0.05**	**0.05**	0.06
**Trapani**							
MI	2.2 ± 0.1	2.2 ± 0.1	2.1±0.1 b	2.3 ± 0.1 a	0.72	**0.03**	0.84
PPI	2.1 ± 0.04	2.3 ± 0.1	2.0 ± 0.01 b	2.4 ± 0.1 a	0.06	**0.00001**	**0.05**
BI	59.9 ± 8.6	63.3 ± 11.2	85.3 ± 5.9 a	37.8 ± 7.9 b	0.74	**0.0001**	0.50
EI	64.9 ± 3.7	69.5 ± 3.9	65.7 ± 5.0	68.7 ± 2.2	0.42	0.61	0.85
SI	63.3 ± 3.0	64.7 ± 2.6	58.0 ± 1.9 b	70.0 ± 2.4 a	0.68	**0.01**	0.78
CI	16.2 ± 2.3	13.5 ± 2.8	13.5 ± 3.0	16.2 ± 2.0	0.48	0.47	0.43
Pp/(B+F)	0.3 ± 0.1	0.3 ± 0.1	0.3 ± 0.1	0.2 ± 0.1	0.71	0.54	0.18

* M, Management; ** Y, year.

## Data Availability

The data presented in this study are available upon request from the corresponding author.

## Supplementary Materials

The following supporting information can be downloaded at: https://www.mdpi.com/article/10.3390/ani12121551/s1. Figure S1: Study areas; Table S1: Detail of soil management (tillage and fertilization applied in the five sites). Table S2: Percentage contribution to the Bray–Curtis dissimilarity in family nematode abundance (SIMPER analysis) among the experimental sites. Mean values and standard errors are reported. Table S3: Percentage contribution to the Bray–Curtis dissimilarity in family nematode abundance (SIMPER analysis) per management (SHD, super-high density; TRAD, traditional) on the whole soil nematode community in the FOG site. Mean values and standard errors are reported. Table S4: Percentage contribution to the Bray–Curtis dissimilarity in family plant-parasitic nematode abundance (SIMPER analysis) per management (SHD, super-high density; TRAD, traditional) on the whole soil nematode community in the RAG site. Mean values and standard errors are reported.
