# Supplementary material for: Impact of Super-High Density Olive Orchard Management System on Soil Free-Living and Plant-Parasitic Nematodes in Central and South Italy
Source: Animals (Basel). 2022 Jun 15;12(12):1551. doi: 10.3390/ani12121551 (PMC9219476; doi:10.3390/ani12121551)
Supplement: Supplementary file 1 [file animals-12-01551-s001.zip › animals-1761536-supplementary.pdf]

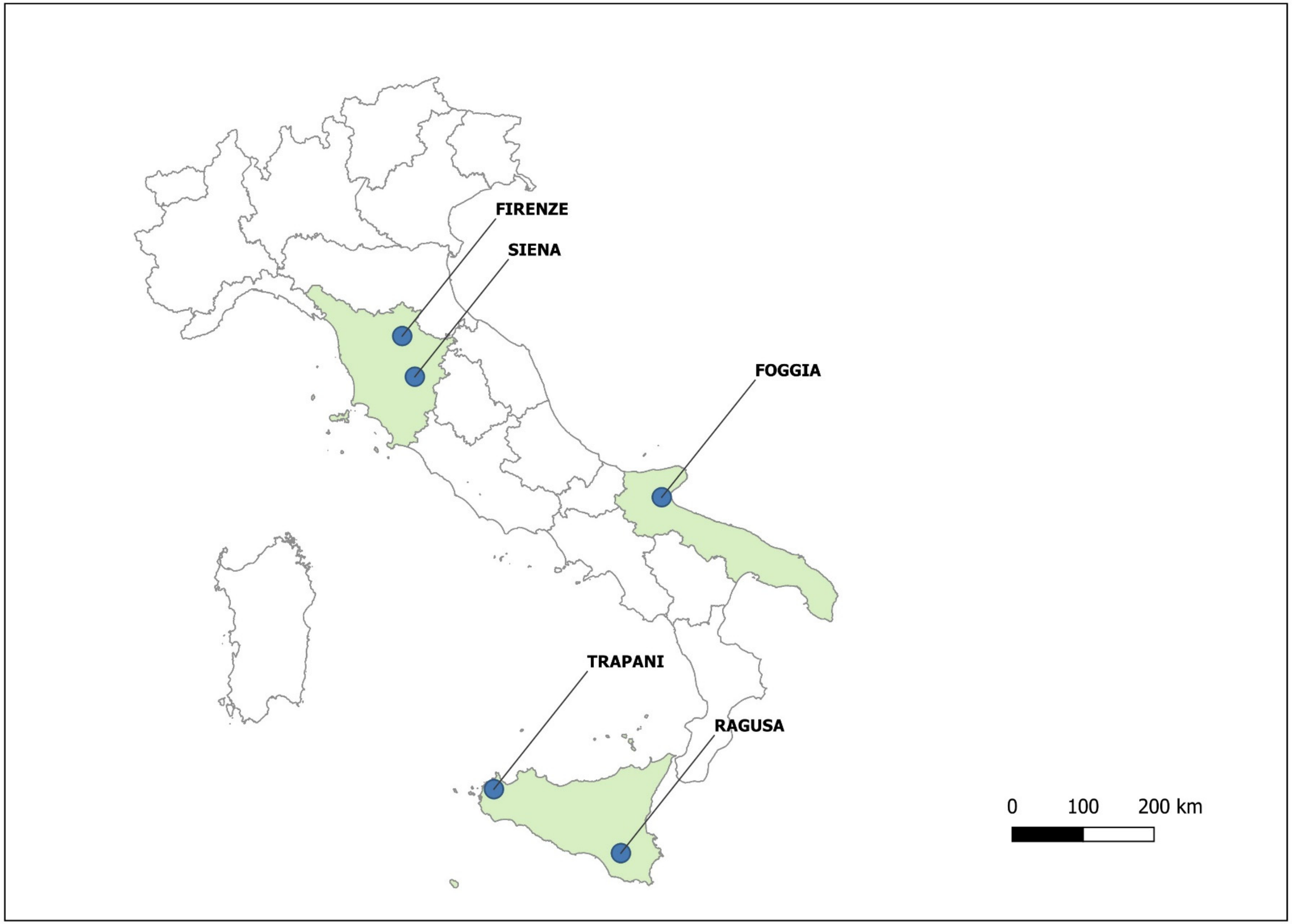

Figure S1. Study areas.

Table S1. Detail of soil management (tillage and fertilization applied in the five sites).

Sites	Soil management	Fertilization
**FIR**	Two tillage using a two-frame grubber with fixed shares in a cultivation depth of 20 cm (Spring and post-harvesting)	Mineral fertilization – NPK 80-50-80 kg/ha<br>Amended fertilization N-organic 3% 1500 kg/ha
**SIE**	Natural green cover, 32 different species in SHD, 26 different species in TRAD	Organic fertilization - Compost BIO-OLIVO 100 kg/ha (N 6%, P_2O_5 3%, K 5%, Organic Carbon 38%)
**FOG**	Two tillage using rotary hoe or harrow with fixed teeth 10-20 cm depth, 3-4 harrowing at 5-10 cm depth	Mineral fertilization - NPK 50-150-100 kg/ha
**RAG**	3-4 tillage by clod breaker 15-20 cm of depth	Organic fertilization – Compost Microzymtrio 400 kg/ha (N 3%, P_2O_5 3%, CaO 9%, SO_3 12%, Fe 3% Organic Carbon 38%)
**TRP**	Green manure with *Vicia faba minor* L. Green manure was mowed and incorporated into the soil in June by rotary tiller and/or rotary hoe, 10-20 cm depth	Organic fertilization – Stable manure 1000 kg/ha

Table S2. Percentage contribution to the Bray-Curtis dissimilarity in family nematode abundance (SIMPER analysis) among the experimental site. Mean values and standard errors are reported.

Taxon	Contribution	Cumulative %	FIR	SIE	FOG	RAG	TRP
Hoplolaimidae	10.05	21.8	13.2 ± 3.11	74.0 ± 13.57	1.0 ± 0.41	134.0 ± 28.58	3.8 ± 0.88
Rhabditidae	7.909	38.94	65.0 ± 14.32	55.6 ± 13.94	38.9 ± 9.60	69.0 ± 13.89	54.3 ± 10.39
Tylenchidae	6.381	52.77	29.0 ± 6.81	24.3 ± 7.65	3.2 ± 0.99	28.7 ± 15.60	28.5 ± 4.45
Cephalobidae	6.037	65.86	26.0 ± 5.57	11.7 ± 2.48	7.4 ± 1.36	43.0 ± 6.36	46.5 ± 5.74
Dorylaimidae	4.595	75.82	23.5 ± 3.30	17.5 ± 2.71	5.8 ± 1.17	29.3 ± 4.82	29.5 ± 2.70
Telotylenchidae	3.336	83.05	6.5 ± 4.69	2.2 ± 1.41	0.3 ± 0.22	1.7 ± 0.90	20.8 ± 5.44
Aphelenchidae	3.056	89.68	7.8 ± 1.41	3.4 ± 0.74	2.5 ± 0.81	5.5 ± 1.12	14.5 ± 3.24
Mononchidae	1.453	92.83	2.9 ± 1.70	0.5 ± 0.19	0.3 ± 0.17	0.7 ± 0.34	1.5 ± 0.55
Paratylenchidae	0.9903	94.97	0	0.3 ± 0.12	1.5 ± 0.80	0.1 ± 0.08	0.8 ± 0.35
Pratylenchidae	0.818	96.75	1.1 ± 0.64	1.8 ± 1.45	0.1 ± 0.1	0.4 ± 0.38	0.3 ± 0.22
Anguinidae	0.635	98.12	0.5 ± 0.46	0.1 ± 0.06	0.8 ± 0.33	0.1 ± 0.08	0.3 ± 0.14
Psilenchidae	0.2645	98.7	0	0	0.2 ± 0.11	0	0.5 ± 0.39
Aphelenchoidae	0.211	99.16	0	0	0.3 ± 0.17	0	0
Discolaimidae	0.1025	99.38	0.1 ± 0.10	0	0	0.1 ± 0.10	0
Meloidogynidae	0.09711	99.59	0	0	0.1 ± 0.06	0	0
Criconematidae	0.06157	99.72	0	0	0	0.1 ± 0.08	0
Heteroderidae	0.04424	99.82	0.04 ± 0.04	0	0	0	0
Longidoridae	0.04392	99.91	0.04 ± 0.04	0	0	0	0
Seinuridae	0.04009	100	0	0	0	0.1 ± 0.08	0
**Overall average dissimilarity**	**46.13%**						

Table S3. Percentage contribution to the Bray-Curtis dissimilarity in family nematode abundance (SIMPER analysis) per management (SHD, super-high density; TRAD, traditional) on the whole soil nematode community in FOG site. Mean values and standard errors are reported.

**Taxon**	**Contribution**	**Cumulative %**	**SHD**	**TRAD**
Rhabditidae	13.81	27.97	13.8 ± 2.77	64.0 ± 16.22
Cephalobidae	6.753	41.65	5.2 ± 1.52	9.7 ± 2.14
Tylenchidae	5.205	52.19	2.9 ± 1.62	3.4 ± 1.20
Aphelenchidae	4.76	61.83	3.0 ± 1.61	2.0 ± 0.35
Dorylaimidae	4.259	70.46	5.7 ± 2.06	5.8 ± 1.21
Paratylenchidae	3.71	77.97	2.7 ± 1.54	0.3 ± 0.14
Hoplolaimidae	3.025	84.1	0.5 ± 0.36	1.4 ± 0.73
Anguinidae	2.105	88.36	0.7 ± 0.44	0.8 ± 0.51
Aphelenchoidae	1.396	91.19	0.3 ± 0.18	0.4 ± 0.29
Mononchidae	1.081	93.38	0	0.6 ± 0.34
Telotylenchidae	0.9185	95.24	0.4 ± 0.42	0.2 ± 0.17
Psilenchidae	0.8511	96.96	0.3 ± 0.22	0
Meloidogynidae	0.7677	98.52	0.2 ± 0.11	0
Pratylenchidae	0.7326	100	0.2 ± 0.17	0.1±0.1
**Overall average dissimilarity**	**49.37%**			

Table S4. Percentage contribution to the Bray-Curtis dissimilarity in family plant-parasitic nematode abundance (SIMPER analysis) per management (SHD, super-high density; TRAD, traditional) on the whole soil nematode community in RAG site. Mean values and standard errors are reported.

**Taxon**	**Contribution**	**Cumulative %**	**SHD**	**TRAD**
Hoplolaimidae	38.53	81.37	171.8 ± 38.75	96.2 ± 40.65
Telotylenchidae	4.335	90.53	0.3 ± 0.26	3.0 ± 1.74
Pratylenchidae	2.393	95.58	0.8 ± 0.75	0
Criconematidae	0.9079	97.5	0.2 ± 0.17	0
Paratylenchidae	0.6392	98.85	0	0.2 ± 0.17
Anguinidae	0.5444	100	0	0.2 ± 0.17
**Overall average dissimilarity**	**47.35%**			
